# Isotherm, Thermodynamic and Kinetic Studies of Elemental Sulfur Removal from Mineral Insulating Oils Using Highly Selective Adsorbent

**DOI:** 10.3390/ma16093522

**Published:** 2023-05-04

**Authors:** Jelena Jankovic, Jelena Lukic, Dejan Kolarski, Djordje Veljović, Željko Radovanović, Silvana Dimitrijević

**Affiliations:** 1 Electrical Engineering Institute Nikola Tesla, University of Belgrade, Koste Glavinica 8a, 11000 Belgrade, Serbia; 2 Faculty of Technology and Metallurgy, University of Belgrade, Karnegijeva 4, 11000 Belgrade, Serbia; 3 Innovation Center of the Faculty of Technology and Metallurgy, Karnegijeva 4, 11000 Belgrade, Serbia; 4 Mining and Metallurgy Institute Bor, Zeleni Bulevar 35, 19210 Bor, Serbia

**Keywords:** elemental sulfur, mineral insulating oil, equilibrium, adsorption isotherms, kinetics, thermodynamic, activation energy, chemisorption

## Abstract

Elemental sulfur (S_8_) is a corrosive sulfur compound which was found to be extremely reactive to silver, causing intensive silver sulfide (Ag_2_S) deposition on on-load tap changer (OLTC) contacts in power transformers. A highly selective adsorbent (HSA), called Tesla’Ssorb, for the removal of S_8_ from mineral insulating oils was prepared from raw material (RM) using the novel procedure. In this study, the adsorption properties of HSA for the removal of S_8_ from the oil were determined. RM and HSA were characterized using various techniques, such as field-emission scanning electron microscopy (FESEM), energy-dispersive X-ray (EDX), and X-ray diffraction (XRD). The performance of HSA was determined by adsorption equilibrium, thermodynamic, and kinetic study through batch experiments, at various temperatures and initial concentrations of S_8_. The obtained results were analyzed by Langmuir and Freundlich adsorption isotherms and it was found that equilibrium data were fitted better with the Langmuir isotherm model. The maximum adsorption capacity was 4.84 mg of S_8_/g of HSA at 353 K. Thermodynamic parameters, such as enthalpy (ΔH°), Gibbs free energy (ΔG°), and entropy (ΔS°), were calculated and it was found that the sorption process was spontaneous (ΔG° < 0) and endothermic in nature (ΔH° > 0). It was found that the adsorption of S_8_ follows pseudo-second-order kinetic model, and the activation energy indicated the activated chemisorption process.

## 1. Introduction

The presence of corrosive sulfur in mineral insulating oils can be the root cause of power transformers and on-load tap changer (OLTC) failures, owing to the formation of electro-conductive sulfides in reaction with power transformer construction metals, copper and silver. The mechanisms of copper sulfide formation from di-benzyl-disulfide (DBDS) have been thoroughly investigated over last decades [1,2,3].

In recent times, cases of copper or silver sulfides formation after oil reclamation have been reported [3,4,5,6,7]. Moreover, the large number of OLTC failures recently reported was connected with the corrosion of silver-plated contacts, after the oil reclamation process, conducted to remove oil ageing products or DBDS. Elemental sulfur (S_8_) was confirmed to be the cause of failure [3,8,9]. In almost all of the cases reported, when silver corrosion was severe and caused by S_8_, copper surfaces were clean, without copper sulfide deposits [4,5,6]. S_8_ may be created as a consequence of the application of used mineral insulating oil regeneration processes with adsorbents, as one of the products of the high-temperature combustion process, during reactivation of the adsorbent. If it remains in oil after reclamation, S_8_ will easily react with OLTC’s silver coated contacts at lower operating temperatures and form conductive silver sulfide deposits during transformer service [1]. Silver sulfide semi-conductive deposits will increase in contact with resistance which will further lead to overheating the OLTC’s contacts. Further, once flaked off and suspended in the transformer, there will be an increase in terms of oil conductivity and the electric stress between the contacts [3,10,11]. Consequently, the dielectric strength of oil will be reduced. Furthermore, electric discharge of the OLTC tap selector contacts in the main tank of the transformer may occur, which may cause a short circuit in the regulating winding [12,13]. The typical concentrations of S_8_, found in mineral oils in service, were up to 10–15 mg/kg [1]. Due to high affinity of S_8_ to react with silver and a large amount of oil in the transformer compared to the small surface of OLTC’s silver plated contacts, only a few mg/kg of S_8_ in the oil is sufficient to create silver corrosion, at low operating temperatures (below 60 °C) [10,14].

One of the most frequently applied temporary mitigation techniques to suppress copper corrosion and the deposition of copper sulfide in the transformer windings is the use of metal passivators, but this has been found to be inefficient for the mitigation of silver corrosion [1,15,16,17].

The mitigation method which provides a permanent solution for the corrosive sulfur problem is oil treatment. It was observed by experience that S_8_ is more complex to remove from the oil than DBDS, which is also corrosive to copper and silver [1,4,5,18,19,20,21]. The solvent extraction process has been proven to be efficient in the removal of corrosive sulfur compounds with a significant reduction of aromatic compounds as well, indicating improvement of oil properties and degree of oil refining [22,23,24,25]. Desulfurization processes with K-PEG or Na-PEG reagent are efficient in the removal of DBDS from the oil, but not for S_8_ removal [26].

Oil treatment processes with adsorbents are based on adsorption or chemisorption of unwanted compounds from mineral oil on the adsorbent surface, by forced circulation of oil thought columns with packed adsorbent bed. Conventional adsorbents, such as alumino-silicates (Fuller’s earth), magnesium aluminium silicate (Ultrasorb), and aluminium oxides (Bauxite), are widely used in oil reclamation processes. They are found to be very efficient in the removal of polar oil ageing products and some corrosive sulfur compounds, namely DBDS. In the case of S_8_ removal, these adsorbents are found to be mostly completely inneficient in the removal of S_8_, especially when S_8_ is present in high concentrations, or of low efficiency when S_8_ was present in low concentrations (up to 10 mg/kg of S_8_) [1,4].

HSA is a tailor-made adsorbent, called Tesla’Ssorb, based on silicon dioxide with bonded silver, specially designed to selectively remove reactive sulfur compound S_8_ (in chemical reaction with deposited silver) in low amounts (up to 3 wt.% of adsorbent) and in short treatment time, in comparison to other adsorbents. The previous study indicated the efficiency of HSA to remove S_8,_ DBDS and oil ageing products, obtaining the oil with restored properties for further use in electrical equipment [27]. Moreover, it was shown that HSA was efficient in the removal of high concentrations of S_8_ (approximately 45 mg/kg) to a final concentration below 1 mg/kg of S_8_, and therefore it can be successfully used to reduce the risks of power transformers failures and to extend transformer life [27].

In this study, the adsorption performance of Tesla’Ssorb by treatment the oil at various temperatures and initial concentrations of S_8_ was investigated. The adsorption process was studied via isotherm, thermodynamic, and kinetic studies. Characterization of the HSA was performed using various analytical methods, in order to define the morphology, phase, and elemental composition of the HSA and to comprehend the adsorption mechanism better.

The kinetic of the adsorption process was studied at three different temperatures, 328, 338, and 353 K. The mechanism of binding of S_8_ molecules to the HSA’s surface was revealed by determining the activation energy for the process from the kinetics data using Arrhenius plots.

## 2. Materials and Methods

### 2.1. Materials and Reagents

Tesla’Ssorb was synthesized by the impregnation method using an adsorbent containing pure silicon dioxide (SiO_2_, 94–98 wt.%) and traces of calcium oxide (CaO, 0.04–1 wt.%) as raw material (RM). The chemicals used for the synthesis of HSA were as follows: silver nitrate (AgNO_3_, extra pure >99.98%, Bor, Serbia), aqueous ammonium hydroxide (25%, Macron, Austria), and water, deionized, H_2_O (Pharma Product, Belgrade, Serbia). Sulfur, powder, reagent grade was procured from Sigma Aldrich (St. Louis, MO, USA), while di phenyl disulfide (DPDS, ≥99%) was obtained from Merck, Germany. The standard solutions and formulated oil samples (spiked with known amount of S_8_) were prepared in used mineral insulating oil free from S_8_ and DPDS. The preparation of oil samples was done with toluene and iso-octane. All chemicals used were of analytical grade and without further purification.

### 2.2. Adsorbent Preparation and Characterization

Tesla’Ssorb for the removal of S_8_ from mineral insulating oils was synthesized by the impregnation method. The procedure comprised of three stages:

1. Annealing the adsorbent support consisted mainly of silicon dioxide (RM) at 150 °C, for 18–24 h, to eliminate adsorbed moisture.
2. Deposition of silver ions (3–6 wt.%) on the adsorbent support by silver nitrate aqueous solution followed by annealing at temperature from 120 °C to 130 °C, during 18–24 h.
3. Deposition of ammonium hydroxide aqueous solution (5–10 wt.%) on the adsorbent support followed by annealing the adsorbent at temperature from 125 °C to 130 °C, during 18–24 h. Ammonium hydroxide is added to neutralize the acidic by-products formed during removal of S_8_ from the oil [28].

For producing an adsorbent with high effectiveness for the removal of sulfur compounds corrosive to silver and to obtain restored oil properties with low acidity for further use in power transformers, both compounds, i.e., silver nitrate and ammonium hydroxide, are necessary [27].

The removal of S_8_ is obtained by the chemical reaction with deposited silver ions on the adsorbent surface, through the chemisorption process, followed by the addition of ammonium hydroxide for the neutralization of acidic by-products. This is shown in the following reaction 1:

(1) $${\mathrm{Ag}}^{+}\;+\;{\mathrm{NO}}_{3}\;+\;S\;+\;{\mathrm{NH}}_{4}{}^{+}\;+\;{\mathrm{OH}}^{-}\;\to \;{\mathrm{Ag}}_{2}S↓\;+\;{\mathrm{NH}}_{4}{}^{+}\;+\;{\mathrm{NO}}_{3}\;+\;H_{2}O$$

An effective binding of silver ions to Tesla’Ssorb silanol groups was achieved with this temperature induced impregnation method. Moreover, the formation of silver oxides was minimized, which may reduce reactivity of silver with corrosive sulfur compounds in the oil. Furthermore, the addition of ammonium hydroxide in a specific and optimal concentration range is necessary for the efficient neutralization of acidic by-products. On the other side, the said compound is not present in an excess amount, since it can reduce the mobility of silver ions for the reaction and the efficiency of corrosive sulfur removal, due to the possible formation of complex salts with silver, i.e., diamine silver (I) [(Ag(NH_3_)_2_]^+^ complex [27].

Basic physical properties of Tesla’Ssorb were analyzed, applying the standard procedures (ASTM, EPA) and in-house validated methods. Field-emission scanning electron microscopy (FESEM) (TESCAN MIRA3XMU, Brno, Czech Republic) operated at 20 keV was used to observe the surface morphology of RM and HSA. An atomic gold layer was deposited on the sample surfaces before analysis. Moreover, energy-dispersive X-ray (EDX) (Oxford Inca 3.2 coupled to a JEOL JSM 5800 scanning electron microscope (JEOL, Akishima, Tokyo, Japan)) analysis was used to analyze the elemental composition of a synthesized adsorbent. X-ray diffraction (Rigaku MiniFlex600 equipped with D/teX Ultra 250 high-speed detector) was performed using CuKα (λ = 1.54 Å), operated at 40 kV and 15 mA, in the 2θ angular range of 3–90°, with 0.02° of step size and 10°/min of scan speed. Measurements were performed using the software MiniFlex Guidance Version 2.1.0.4. The mineral identification was performed in the software PDXL 2 Version 2.4.2.0 and the obtained diffractograms were compared with the data from the database ICDD (PDF-2 Release 2015 RDB).

### 2.3. Preparation and Analysis of S_8_ in Mineral Insulating Oils

Solutions of S_8_ were prepared by dissolving a necessary amount of sulfur powder in used mineral insulating oil free from S_8_, in order to obtain the desired concentrations of S_8_ ranging from approximately 80 to 150 mg/kg. The concentrations of S_8_ in used mineral oils were determined using GC-ECD (Thermo Scientific 1300 system with an autosampler AI 3000 series, Milan, Italy), according to IEC TR 62697-3.

### 2.4. Batch Adsorption Experiments

**Figure 1:**
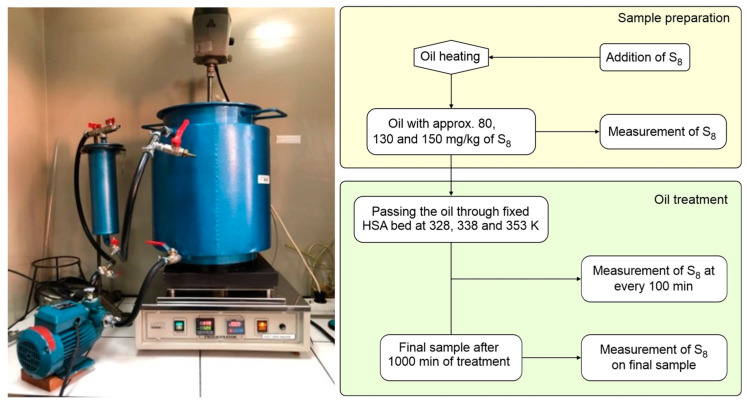
Pilot scale system for oil reclamation (**left**) and schematic diagram of the experiments (**right**).

The batch adsorption experiments were evaluated in terms of the effect of initial S_8_ concentrations, contact time, and temperature, in order to study adsorption isotherms, adsorption kinetics, and thermodynamic. Experiments were carried out using the percolation process, on a pilot scale reclamation unit with a digital temperature controlled hot plate (Figure 1). The 17.3 kg of used mineral insulating oil, spiked with various initial concentrations of S_8_ from approximately 80 to 150 mg/kg was pumped through a stainless-steel column filled with 523 gr of HSA (i.e., 3 wt.% of adsorbents compared to the mass of oil) at a flow rate of ca. 200 mL/min. To achieve equilibrium at constant temperatures of 328, 338, and 353 K respectively, a contact time of 1000 min was fixed in all experiments. At predetermined times, the samples were collected and analyzed.

The percentage of S_8_ adsorption (%) and adsorption capacities (q_e_ and q_t_) were calculated using Equations (2)–(4):

(2) $$\%adsorption=\frac{(C_{0}-C_{e})100}{C_{0}}$$

(3) $$q_{e}=\frac{(C_{0}-C_{e})m_{oil}}{m_{ads}}$$

(4) $$q_{{}_{t}}=\frac{(C_{0}-C_{t})m_{oil}}{m_{ads}}$$

where *q_e_* is the amount of adsorbate adsorbed at equilibrium per unit weight of the adsorbent (mg g^−1^), *q_t_* is the amount of adsorbate adsorbed at any time (mg g^−1^), *C*_0_ and *C_e_* are the initial and equilibrium concentrations of S_8_ (mg·kg^−1^), m_oil_ is the mass of oil (kg), and *m_ads_* is the adsorbent mass (g).

In order to determine the kinetic model, limiting level, and mechanism of the adsorption of S_8_ onto the HSA, the pseudo-first-order [29], pseudo-second-order [30], and intraparticle diffusion kinetic models were used [31]. The Langmuir [32] and Freundlich [33] models were used to describe the equilibrium properties of S_8_ adsorption on HSA. The thermodynamic parameters, such as the change of Gibbs free energy (Δ*G_o_°*), enthalpy (Δ*H_o_°*), and entropy (ΔS_o_°) were calculated in order to confirm the reaction mechanism (reaction 1). Insight into the binding process was provided by the determination of activation energy using the Arrhenius plot.

## 3. Results and Discussion

### 3.1. Characterization of Tesla’Ssorb

The Tesla’Ssorb was characterized by determining some of its general physical properties, e.g., pH, flash point, melting point, and bulk density, employing standard methods. The results are shown in Table 1.

Based on the results of textural characteristics of HSA, such as specific surface area, S_BET_ (29.28 m^2^/g), total pore volume, V_total_ (0.2026 cm^3^/g), mesopore volume, V_meso_ (0.2025 cm^3^/g), average pore diameter, D_A_ (19.2 nm), and pore size distribution, D_BHJ_ (25.1 nm), reported in previous work, HSA was classified as a mesoporous material [27].

**Figure 2:**
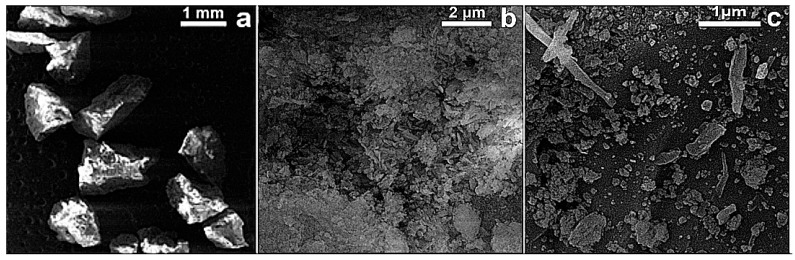
FESEM images of RM (before impregnation of Ag^+^ ions)—at different magnifications: (**a**) 37×; (**b**) 20 kx and (**c**) 50 kx.

**Figure 3:**
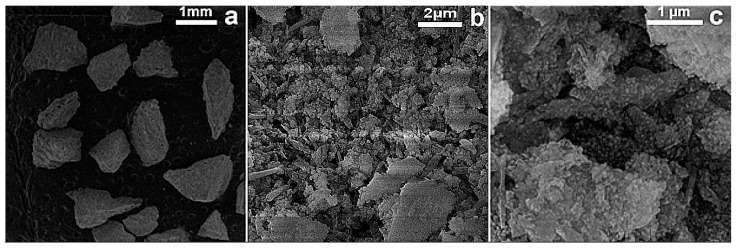
FESEM images of Tesla’Ssorb (after impregnation of Ag^+^ ions)—at different magnifications: (**a**) 37×; (**b**) 20 kx and (**c**) 50 kx.

The FESEM micrographs of RM (before) and Tesla’Ssorb (after) modification, i.e., impregnation and sorption of Ag ions, are displayed in Figure 2 and Figure 3.

Based on the FESEM surface analysis of the RM (before) and Tesla’Ssorb (after) sorption of Ag ions at lower magnifications, irregularly shaped agglomerates with dimensions larger than 1 mm are observed (Figure 2a). At higher magnifications, it was observed that both RM and Tesla’Ssorb agglomerates consist of rod-like particles and particles of irregular shape (Figure 2b,c and Figure 3b). In the case of the Tesla’Ssorb, rod-like particles were found to be more elongated than the primary RM particles. Further analysis of Tesla’Ssorb at the highest magnifications shows the existence of nano-sized spherical clusters on the surface of the primary particles (Figure 3c). This phenomenon has not been observed in the morphology of the RM (Figure 2c). Therefore, it can be concluded that these spherical clusters, with a diameter of approximately 60 nm, are silver nanoparticles formed during the modification of the RM, by silver nitrate impregnation.

**Figure 4:**
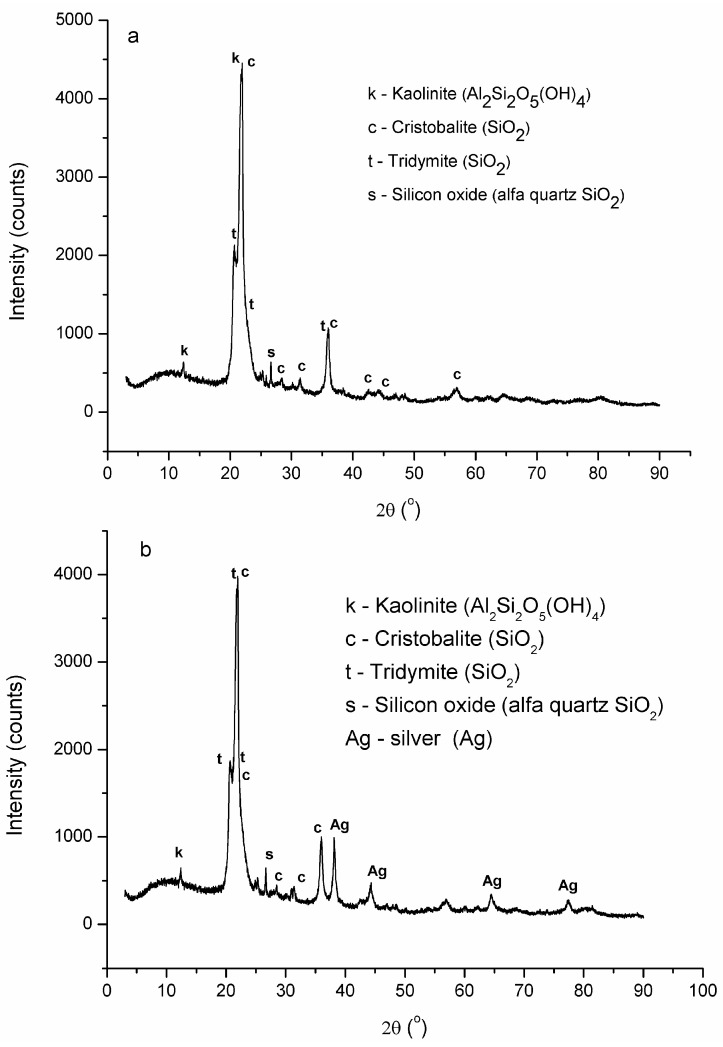
X-ray diffraction patterns of: (**a**) RM; (**b**) Tesla’Ssorb.

The X-ray diffraction patterns of RM and Tesla’Ssorb are shown in Figure 4a,b. Both samples showed reflections of tridymite, SiO_2_ (COD 901-3493), kaolinite, Al_2_Si_2_O_5_(OH)_4_, and cristobalite, SiO_2_ (COD 900-8230) in larger quantities, and quartz, SiO_2_ in very small quantities. Furthermore, the Tesla’Ssorb diffractogram showed a very sharp peak at 2θ = 38.1 Å and low-intensity broad peaks at 44, 64, and 77 Å (Figure 4b), in comparison with RM (Figure 4a), which confirmed the presence of silver in Tesla’Ssorb.

**Figure 5:**
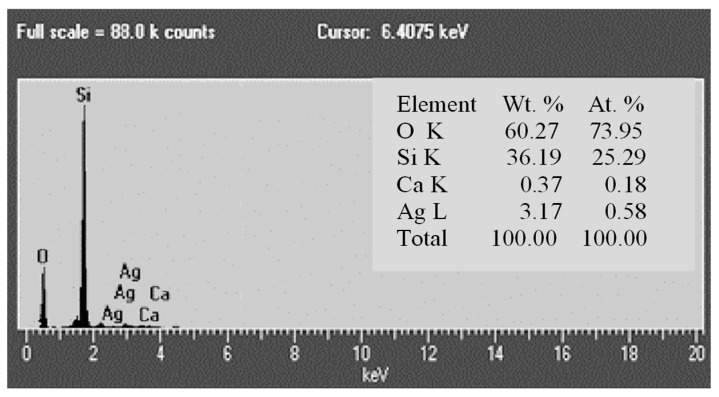
EDS analysis of the chemical composition of Tesla’Ssorb.

EDS analysis of the chemical composition of Tesla’Ssorb also confirmed the presence of incorporated silver ions (Figure 5). The major constituents of the adsorbent are silicon (36.19 wt.%) and oxygen (60.27 wt.%), which corresponds to the chemical formula (SiO_2_), with 3.17 wt.% of Ag and a minor amount of Ca (0.37 wt.%).

### 3.2. Effect of Initial S_8_ Concentration

**Figure 6:**
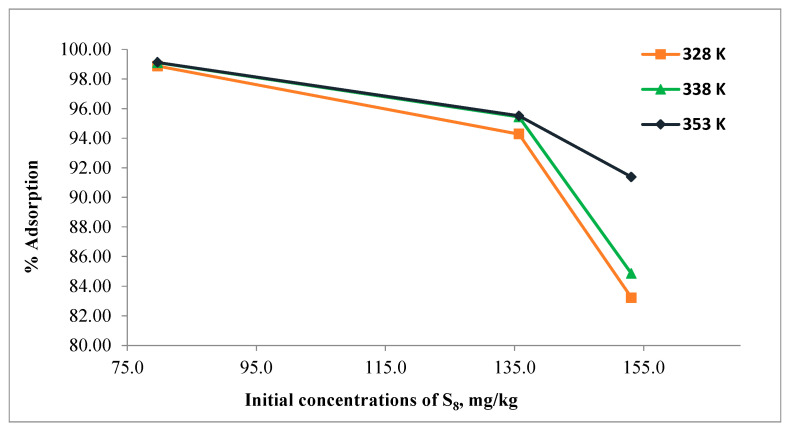
Effect of initial concentration of S_8_.

The effect of initial S_8_ concentration in the range of 79.7–153.1 mg/kg on adsorption using Tesla’Ssorb was carried out at different temperatures of 328, 338, and 353 K, and the results are given in Figure 6.

Due to the fixed amount of adsorbent used in this study (3 wt.%), it can be seen from the Figure 6 that the percentage of S_8_ adsorption decreased as the initial concentration of S_8_ increased. The presence of more molecules of S_8_ in the oil per unit number of adsorbent sites (with bonded silver ions) leads to a saturation of the adsorption sites.

For the 79.7 mg/kg of S_8_, the percentage of adsorption was found to be approximately 99.0% at all temperatures, while for 153.1 mg/kg of S_8_, the relevant values were 83.2%, 84.8%, and 91.4% at 328, 338, and 353 K respectively.

Moreover, the higher amount of S_8_ is adsorbed by Tesla’Ssorb at the highest initial concentrations of S_8_ (153.1 mg/kg). The initial concentration of S_8_ has a significant impact on the adsorption capacity since the concentration gradient provides the necessary driving force for adsorption between the oil and the Tesla’Ssorb surface.

The increase in initial S_8_ concentration improved the interaction between incorporated silver ions on the Tesla’Ssorb and S_8_ molecules as well as the adsorption capability of Tesla’Ssorb to remove S_8_ from the oil, even if S_8_ is present in the oil in very high concentrations.

### 3.3. Effect of Contact Time

**Figure 7:**
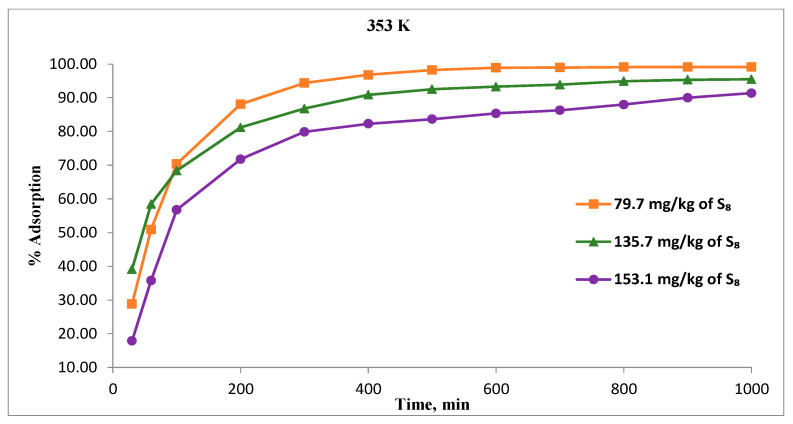
Effect of contact time on the S_8_ removal for different concentrations at 353 K.

Figure 7 illustrates the relation between contact time and S_8_ adsorption on Tesla’Ssorb at various initial S_8_ concentrations at highest temperature (353 K). The adsorption was very fast during the first 300 min. With a further increase of time, the rate of adsorption decreased, and the adsorption equilibrium was achieved within 600 min, i.e., within 1000 min (for the highest concentration of S_8_, 153.1 mg/kg), as shown in Figure 7.

At adsorption equilibrium, a constant value of S_8_ is obtained, where no more S_8_ is removed from the oil. These results are in very good correlation with the equilibrium adsorption capacity of Tesla’Ssorb previously reported, for the treatment of oil from a real 35 kV power transformer, performed on-site [27].

The fast initial adsorption is due to the increased concentration gradient between the S_8_ in the oil and incorporated silver ions on the adsorbent support, since there are a large number of unoccupied sites available initially. Achieving the adsorption equilibrium over time is due to the reduced number of available adsorbent sites for reaction with S_8_ molecules and the low concentration of S_8_ in the oil. At the highest adsorption temperature of 353 K, the percentage of adsorption at equilibrium stage decreased from 99.1% to 91.4% with increasing S_8_ concentration from 79.7 to 153.1 mg/kg.

### 3.4. Effect of Temperature

**Figure 8:**
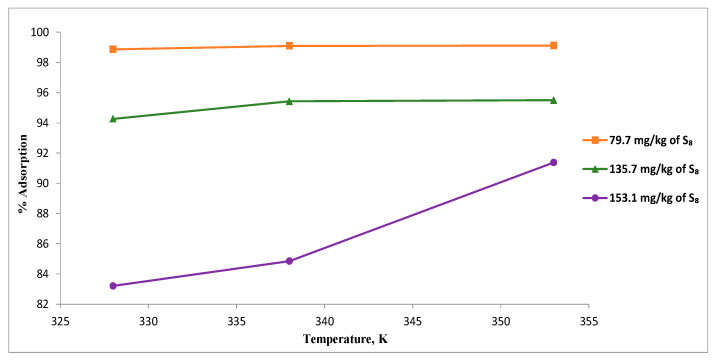
Effect of temperature on the S_8_ removal.

The removal of S_8_ using Tesla’Ssorb was studied as a function of temperature, with experiments on oils containing different initial S_8_ concentrations of (79.7 to 153.1) mg/kg, at different temperatures of (328, 338, and 353) K using a fixed adsorbent dosage of 3 wt.% relative to the mass of oil (Figure 8).

At the lowest temperature (328 K), a significant difference in percentage of adsorption was observed, for various initial concentrations of S_8_ (79.7, 135.7 and 153.1) mg/kg in comparison with results obtained at 353 K, where the degree of S_8_ removal was more than 90% (from 91.4% to 99.1%), for the same initial concentrations of S_8_.

These results indicated that higher temperatures increase the activity of incorporated silver ions on the Tesla’Ssorb to react with S_8_ molecules present in the oil, promoting the removal of S_8_ from the oil in a wide range of concentrations. Therefore, it can be concluded that 353 K can be used as an optimal temperature for S_8_ removal for the oil, in a wide range of concentrations.

Results presented herein showed that the adsorption capacity of Tesla’Ssorb was enhanced as the temperature was increased, indicating the endothermic nature of the S_8_ adsorption process.

### 3.5. Adsorption Isotherm Study

The experimental data were analyzed using Langmuir and Freundlich isotherms to fully understand the type of interaction between the adsorbate and the adsorbent.

**Figure 9:**
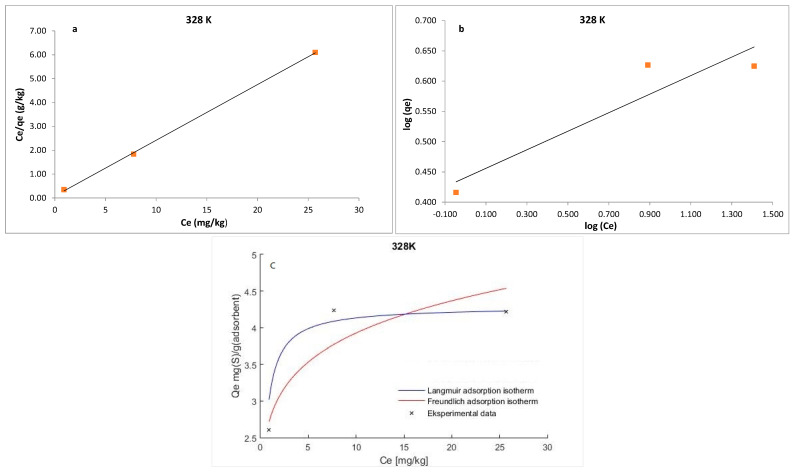
Linearized: (**a**) Lanqmuir; (**b**) Freundlich isotherm models and (**c**) Non-linearized models for S_8_ adsorption at 328 K (adsorption conditions: C_0_, 79.7–153.1 mg/kg; 3 wt.% of Tesla’Ssorb).

**Figure 10:**
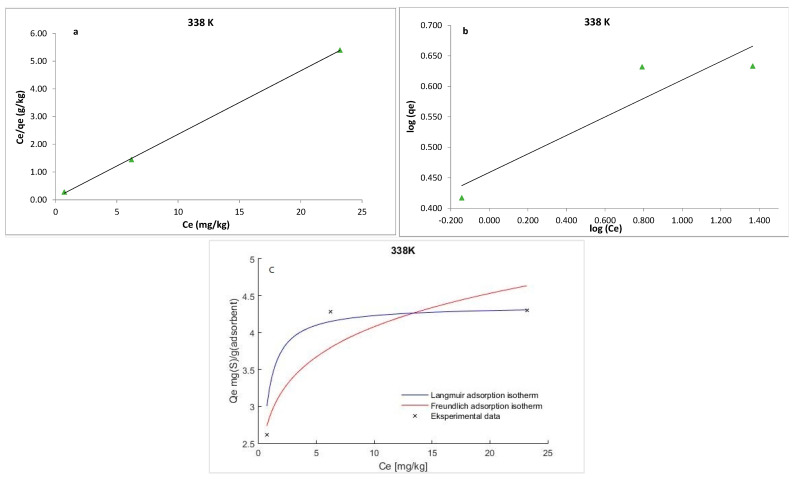
Linearized: (**a**) Lanqmuir; (**b**) Freundlich isotherm models and (**c**) Non-linearized models for S_8_ adsorption at 338 K (adsorption conditions: C_0_, 79.7–153.1 mg/kg; 3 wt.% of Tesla’Ssorb).

**Figure 11:**
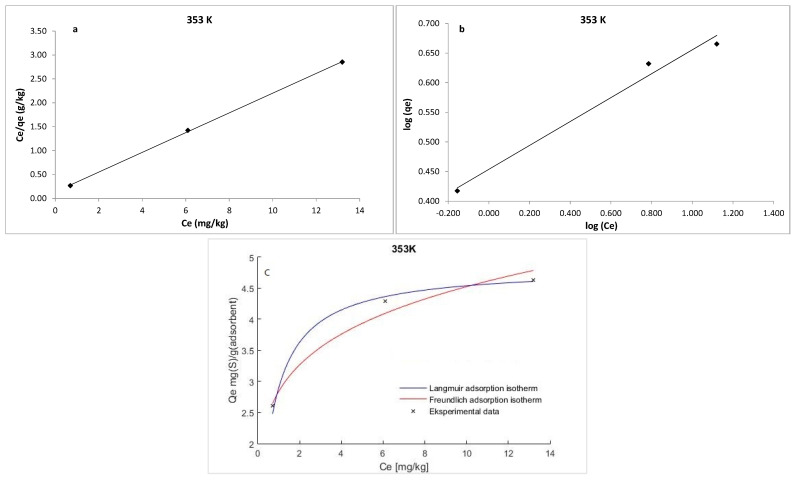
Linearized: (**a**) Lanqmuir; (**b**) Freundlich isotherm models and (**c**) Non-linearized models for S_8_ adsorption at 353 K (adsorption conditions: C_0_, 79.7–153.1 mg/kg; 3 wt.% of Tesla’Ssorb).

The Langmuir isotherm model is based on the premise that the adsorbent is structurally homogeneous, covered in a monolayer with no interaction between the molecules of the absorbate. The equation developed by Langmuir is expressed as follows:

(5) $$\frac{C_{e}}{q_{e}}=\frac{1}{q_{m}K_{L}}+\frac{C_{e}}{q_{m}}$$

where *q_e_* is the amount of S_8_ adsorbed at equilibrium per unit weight of the adsorbent (mg·g^−1^), *C_e_* is the equilibrium concentrations of S_8_ (mg·kg^−1^) in the oil, *K_L_* is the Langmuir constant (kg·mg^−1^), and *q_m_* is the maximum adsorption capacity (mg·g^−1^). The maximum adsorption capacity (*q_m_*) of Tesla’Ssorb and Langmuir constant were determined from the curves *C_e_/q_e_* vs. 1/*C_e_* (Figure 9, Figure 10 and Figure 11). It was found that the maximum adsorption capacity of Tesla’Ssorb is 4.84 mg·g^−1^, obtained at 353 K (Table 2).

In comparison with Langmuir isotherm model, the Freundlich isotherm presumes the interaction between adsorbed molecules on heterogeneous surfaces. This isotherm is expressed as follows:

(6) $$\log q_{e}=\log K_{F}+\frac{1}{n}\log C_{e}$$

where *K_F_* and *n* are Freundlich constants related to the adsorption capacity and adsorption intensity, respectively. The typical parameters for the Langmuir and Freundlich isotherm models and R^2^ values at three temperatures are given in Table 2.

The separation factor (*R_L_*) can be used to describe the Langmuir isotherm’s feasibility and may be calculated using the following equation:

(7) $$R_{L}=\frac{1}{\left(1+K_{L}C_{0}\right)}$$

The *R_L_* value defines the form of isotherm and the adsorption process nature, which is irreversible (*R_L_* = 0), unfavorable (*R_L_* > 1), linear (*R_L_* = 1), and favorable (0 < *R_L_* < 1). The obtained *R_L_* values in this study were in the range of 0−1, thus confirming that the Tesla’Ssorb is a suitable adsorbent for the adsorption of S_8_ under defined testing conditions.

Graphical presentations for Langmuir and Freundlich equilibrium isotherms of S_8_ adsorption onto Tesla’Ssorb (linear and non-linear models) are given in Figure 9, Figure 10 and Figure 11. Correlation coefficients (R^2^ values) were used to compare the suitability of used isotherm models. Since the obtained R^2^ values for Langmuir isotherm model were higher in comparison with Freundlich isotherm (Table 2), it can be concluded that the Langmuir isotherm fits better. The experimental data, in both linearized and non-linearized fitting curves, were fitted better to the Langmuir isotherm model, as shown in Figure 9, Figure 10 and Figure 11. Furthermore, it can be observed that maximum adsorption capacity (*q_m_*) increases with increasing temperature, due to the endothermic nature of adsorption. This was further confirmed by the analysis of thermodynamic parameters.

### 3.6. Adsorption Thermodynamics

The thermodynamic parameters of elemental sulfur removal from mineral insulating oils using Tesla’Ssorb provide information about: the adsorption process (whether it is spontaneous or not), the nature of interactions between the Tesla’Ssorb and S_8_ molecules, and the conditions for reaction which will provide the highest rate of S_8_ removal. The adsorption mechanism can be determined though thermodynamic parameters, such as change in Gibb’s free energy (Δ*G*°), enthalpy of adsorption (Δ*H°*), and entropy (Δ*S*°). The change in the Gibbs free energy (Δ*G*°) was calculated from the distribution coefficient (Table 3). The relationship is shown in Equation (8), as follows:

(8) $$\Delta G=-RT\ln K_{d}$$

where Δ*G* is the calculated change in the Gibbs free energy, *R* is the universal gas constant (8.314 J·mol^−1^·K^−1^), and *T* is the absolute temperature in Kelvin, respectively.

*K_d_* is the distribution coefficient which can be calculated from the concentration of S_8_ adsorbed at equilibrium (*q_e_*) and S_8_ concentration in oil at equilibrium (*C_e_*), as follows:

(9) $$K_{d}=\frac{q_{e}}{C_{e}}$$

The relationship of (Δ*G*) to enthalpy change (Δ*H*) and entropy change (Δ*S*) of adsorption as well as the relationship between (Δ*G*) and the (ln*K_d_*) are shown in Equations (10) and (11), respectively:

(10) ΔG=ΔH−TΔS

Substituting Equation (8) into Equation (10) gives the Equation (11): 

(11) $$\ln K_{d}=\frac{\Delta S}{R}-\frac{\Delta H}{RT}$$

**Figure 12:**
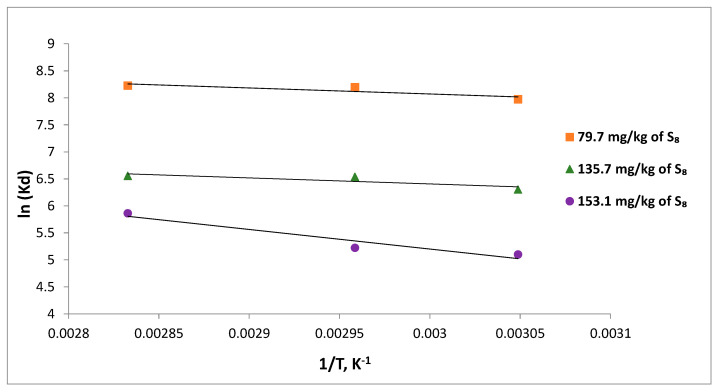
Thermodynamic plots for the adsorption of S_8_ on Tesla’Ssorb.

The values of Δ*H* and Δ*S* are determined from the slope and the intercept of the linear plot of ln*K_d_* vs. 1/*T* respectively. The plots of lnK_d_ vs. 1/*T*, calculated at three temperatures, 328, 338, and 353 K, are shown in Figure 12.

The negative values of Gibbs free energy for S_8_ adsorption indicated that the process was spontaneous, and more spontaneous and thermodynamically favorable at higher temperatures, for various initial concentrations of S_8_.

Physical adsorption is indicated by ΔG values between −20 (kJ·mol^−1^) up to 0 (kJ·mol^−1^) while ΔG values between −80 and −200 (kJ·mol^−1^) suggest a potential chemisorption process. The calculated values shown in Table 3 suggest an adsorption based on combined physical adsorption and chemical reaction, i.e., chemisorption process [34].

The positive ΔH values determined for all initial S_8_ concentrations have indicated that the reaction is endothermic in nature. The randomness at the solid-liquid boundary layer during the adsorption can be described by standard entropy changes, ΔS. The ∆S values were positive for all initial concentrations, which indicated an increase in randomness of the solid–liquid phases at the boundary layer and a high affinity and probability of reaction between the S_8_ in mineral oil and metal ions on Tesla’Ssorb.

### 3.7. Adsorption Kinetics

Pseudo-first-order, pseudo-second-order, and Weber–Morris intraparticle diffusion models were used to analyze the adsorption kinetics of S_8_ on Tesla’Ssorb.

A linear form of pseudo-first model is:

(12) $$\log(q_{e}-q_{t})=\log q_{e}-\frac{k_{1}}{2.303}t$$

where *k*_1_ is the pseudo-first order kinetics rate constant (min^−1^), calculated from the linear plots of log(*q_e_* − *q_t_*) vs. *t*.

Pseudo-second order has a linear form as follows:

(13) $$\frac{t}{q_{t}}={\frac{1}{k_{2}q_{e}{}^{2}}}^{}+\frac{1}{q_{e}}t$$

where *k*_2_ is the pseudo-second order kinetics rate constant (g mg^−1^ min^−1^).

The Weber–Morris intraparticle diffusion model investigates the intraparticle uptake of adsorbate and the pore diffusion in adsorption. The linear form of this model is:

(14) $$q_{t}=k_{id}t^{\frac{1}{2}}+C$$

where *k_id_* (mg/g min^1/2^) is the intraparticle diffusion adsorption rate constant, and *C* is a parameter related to the boundary layer’s thickness.

Calculated kinetic parameters summarized in Table 4 for different initial S_8_ concentrations at different temperatures indicated that the data fitted to the pseudo-second-order model provided a higher value of correlation coefficient R^2^ (from 0.9972 to 0.9999) in comparison to the pseudo-first-order and intra-particle diffusion kinetic models. Moreover, the calculated equilibrium adsorption capacity (*q_e,cal_*) from pseudo-second-order kinetics is closer to the experimental value (*q_e,exp_*). This is also in line with calculated values of maximum adsorption capacities of Tesla’Ssorb (*q_m_*) at different temperatures using the Langmuir isotherm (Table 2). This implies that the adsorption may be governed by chemical adsorption.

**Figure 13:**
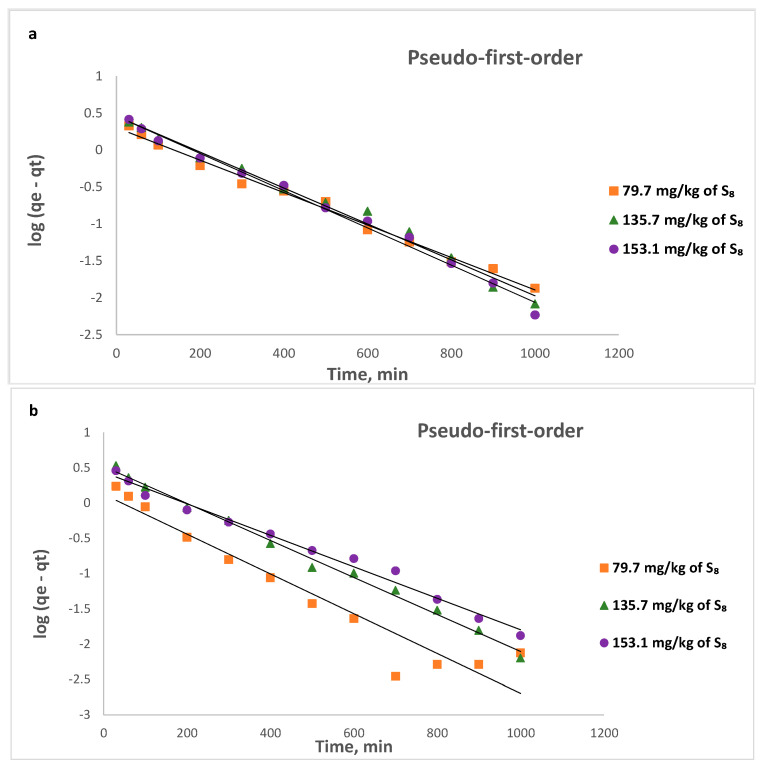

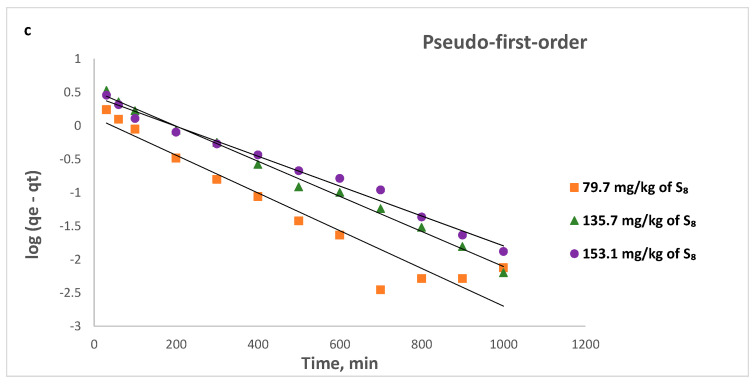
Pseudo-first- order linear kinetic plots for the removal of S_8_ at: (**a**) 328 K; (**b**) 338 K; (**c**) 353 K.

**Figure 14:**
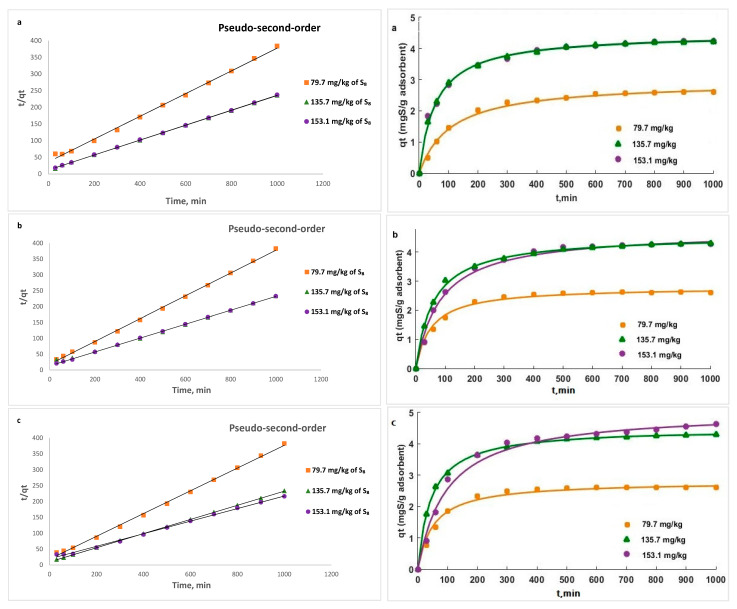
Pseudo-second-order linear (**left**) and non-linear (**right**) kinetic plots for the removal of S_8_ at: (**a**) 328 K; (**b**) 338 K; (**c**) 353 K.

**Figure 15:**
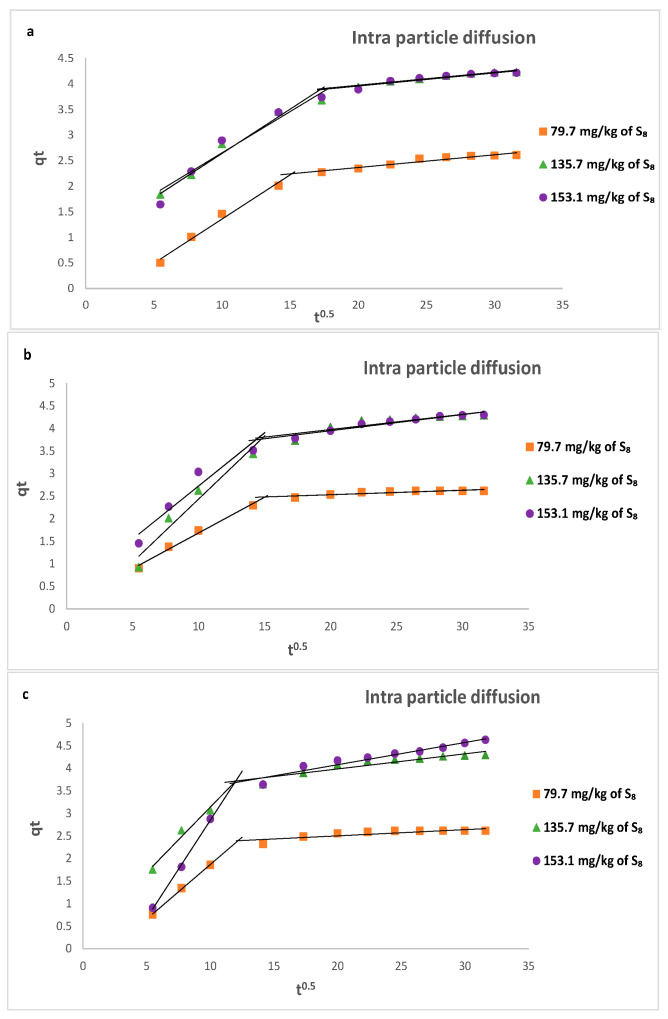
Intra-particle diffusion linear kinetic plots for the removal of S_8_ at: (**a**) 328 K; (**b**) 338 K; (**c**) 353 K.

The graphical presentations for pseudo-first, pseudo-second order kinetic (linear and non-linear), and intra-particle diffusion models at three different temperatures are given in Figure 13, Figure 14 and Figure 15 respectively.

The operating conditions, such as temperature and initial concentration of S_8_, have the influence of the value of k_2_. This decreases as the initial S_8_ concentration increases, since it takes a longer to reach equilibrium (Figure 14). At higher concentrations, lower adsorption rates are obtained, due to high competition for the Tesla’Ssorb surface active sites.

The rate controlling steps that occurred during the adsorption process were determined using the Weber–Morris model. Figure 15 shows that the plot was not linear over the whole time range and had two linear segments, indicating that intra-particle diffusion was not the only rate-controlling step and that the sorption process is more complex and involves several diffusion resistances.

At all temperatures, the slope of the first portion was higher than the slope of the second portion, indicating that the S_8_ molecules diffused more quickly through the boundary layer than through the pores of the adsorbent. Initially, the S_8_ molecules were rapidly adsorbed on the surface of the Tesla’Ssorb due to chemical interactions between S_8_ and incorporated Ag ions on active sites. Later on, after saturation was reached, the S_8_ molecules diffused into the internal surfaces of the adsorbent particles, further followed by the establishment of equilibrium.

### 3.8. Activation Energy (E_a_)

The activation energy (*E_a_*) determines the correlation and dependence of reaction between S_8_ and silver with temperature. In absorption processes, *E_a_* is the minimum amount of energy needed for interaction between the adsorbate, i.e., elemental sulfur and incorporated silver ions in Tesla’Ssorb.

The activation energy, *E_a_* (kJ mol^−1^) for S_8_ adsorption was determined from the Arrhenius equation, at three different temperatures (328, 338 and 353) K. The Arrhenius plot was developed by plotting the lnk_2_ vs. 1/T (in Kelvin). The Arrhenius equation’s linearized form is provided in Equation (15):

(15) $$\ln k_{2}=\ln A-\frac{E_{a}}{RT}$$

where *k*_2_ is the pseudo-second-order rate constant (g·mg^−1^·min^−1^), *E_a_* is the activation energy of adsorption (kJ mol^−1^), and *A* is the Arrhenius constant.

**Figure 16:**
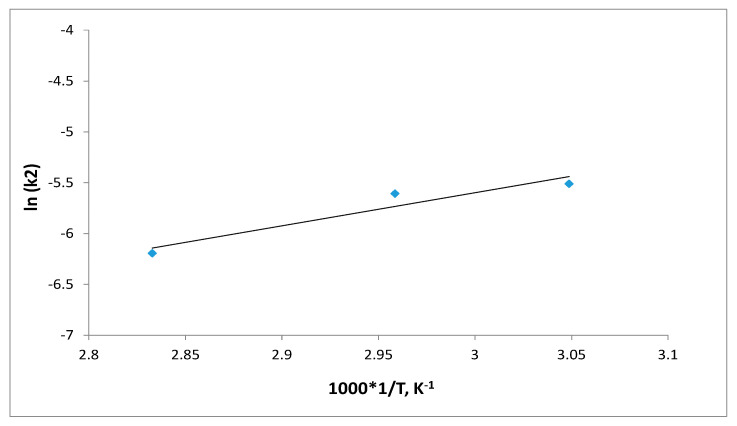
Activation energy plot for S_8_ adsorption.

The value of *E_a_* defines the type of adsorption, which may be physical or chemical. The activation energy for physical adsorption is usually no higher than 4.2 kJ mol^−1^, due to the weak forces that occur in physical adsorption. Chemical adsorption is more specific and involves much stronger forces than in physical adsorption. There are two types of chemical adsorption, activated and non-activated as not frequently present. According to a finite activation energy in the Arrhenius equation (8.4–83.7 kJ mol^−1^), activated chemical adsorption occurs when the rate changes with temperature. The rapid occurrence of a non-activated chemisorption suggests that the activation energy is close to zero [35]. A linear plot between lnk_2_ and 1/T shows a straight line (Figure 16). The activation energy (*E_a_*) for the removal of the highest concentration of S_8_ (153.1 mg/kg) was found to be 27.1 kJmol^−1^, suggesting that S_8_ adsorption by Tesla’Ssorb is controlled by an activated chemical adsorption.

The obtained result of activation energy is in good correlation with thermodynamic parameters, thereby confirming the postulated mechanism of chemical reaction (Reaction 1) and high affinity, i.e., reactivity of molecules S_8_ and silver.

## 4. Conclusions

The adsorption performance of new mesoporous material with spherical clusters of silver nanoparticles, HSA-Tesla’Ssorb, was investigated in this study. The Tesla’Ssorb was synthesized, characterized, and tested for the removal of S_8_ in wide range of concentrations (from 79.7 to 153.1 mg/kg). The removal of sulfur from the mineral oil is obtained by chemical reaction of Ag ions (deposited on the Tesla’Ssorb surface), with sulfur, followed by neutralization of acidic by-products with ammonia. FESEM, EDS, and XRD analyses confirmed the presence of incorporated silver in Tesla’Ssorb, which is essential in the removal of S_8_ from the mineral oils.

The results of adsorption experiments on a pilot scale unit, in terms of the effect of contact time and equilibrium absorption capacity of Tesla’Ssorb, match very well with the results previously reported. This confirms that the pilot scale unit is a very good tool for predicting an adsorbent’s on-site performance, even if a high concentration of S_8_ is present in the oil (153.1 mg/kg). The effect of temperature was pronounced at the highest initial concentrations of S_8_, confirming that 353 K can be used as an optimal temperature for the efficient removal of S_8_ from the oil, in a wide range of concentrations.

Experimental results agree very well with the Langmuir adsorption isotherm model. The thermodynamic parameters from the study indicated that the adsorption of the S_8_ is spontaneous and endothermic.

According to the kinetics studies, it was observed that the adsorption of S_8_ is very fast in the beginning and decreases towards approaching the equilibrium. Kinetics data were best fitted by the pseudo-second order model, indicating that the rate-limiting step in the adsorption process is chemisorption.

The results of the intra particle diffusion kinetic model indicated that the adsorption process could be divided into two stages. The fast adsorption in the first stage was driven by boundary layer diffusion while the slow adsorption in the second stage was attributed to intraparticle diffusion where the equilibrium adsorption was achieved.

The activation energy (*E_a_*) calculated from Arrhenius equation also indicated activated chemical adsorption of S_8_ onto Tesla’Ssorb. Compared to various conventional adsorbents used in oil reclamation processes, a low amount (up to 3 wt.%) of Tesla’Ssorb was verified to have high capacity to remove variable concentrations of S_8_ from mineral insulating oils in a short treatment time.

## Figures and Tables

**Table 1:** Characteristics of Tesla’Ssorb.

Parameter	Value	Method Used
pH	6.94	EPA 9045D
Melting point (°C)	>1600	In-house
Flash point (°C)	>500	In-house
Bulk density (kg/m^3^)	730	ASTM C29

**Table 2:** Adsorption isotherm data for S_8_ on Tesla’Ssorb.

Parameters	328 K	338 K	353 K
Langmuir Isotherm Constants			
*q_m_* (mg·g^−1^)	4.29	4.37	4.84
*K_L_* (kg·mg^−1^)	2.62	3.06	1.50
R^2^	0.9996	0.9997	0.9997
*R_L_*	0.0048	0.0041	0.0083
Freundlich Isotherm Constants			
*K_F_* (mg·g^−1^) (kg·mg^−1^)^1/n^	2.76	2.88	2.84
*n*	6.53	6.59	4.95
R^2^	0.8711	0.8621	0.9825

**Table 3:** Thermodynamic parameters for S_8_ adsorption on Tesla’Ssorb.

S_8_ conc. (mg/kg)	Δ*H°* (kJ·mol^−1^)	Δ*S°* (J·mol^−1^ K^−1^)	Δ*G°* (kJ·mol^−1^)		
			328 K	338 K	353 K
79.7	9.28	94.93	−21.7	−23.0	−24.1
135.7	9.27	81.05	−17.2	−18.4	−19.2
153.1	30.08	133.47	−13.9	−14.7	−17.2

**Table 4:** Kinetic parameters for S_8_ adsorption.

T, K	S_8_ conc. (mg/kg)	Pseudo-First-Order				Pseudo-Second-Order			Intra-Particle Diffusion		
		q_e_,exp (mg·g^−1^)	q_e_,cal (mg·g^−1^)	k_1_ (min^−1^)	R^2^	q_e_,cal (mg·g^−1^)	k_2_ (g·mg^−1^·min^−1^)	R^2^	k_id_ (mg·g^−1^·min^−1/2^)	C (mg·g^−1^)	R^2^
328 K	79.7	2.62	2.00	0.0051	0.9920	2.93	0.0032	0.9977	0.0729	0.6292	0.8430
	135.7	4.24	2.88	0.0055	0.9880	4.48	0.0040	0.9997	0.0875	1.8420	0.8710
	153.1	4.22	2.87	0.0058	0.9912	4.47	0.0040	0.9999	0.0883	1.8179	0.8466
338 K	79.7	2.62	1.33	0.0064	0.9224	2.78	0.0071	0.9990	0.0576	1.1024	0.7717
	135.7	4.29	3.29	0.0060	0.9939	4.57	0.0037	0.9999	0.1109	1.3124	0.8123
	153.1	4.31	2.73	0.0051	0.9873	4.57	0.0037	0.9999	0.0931	1.7611	0.8234
353 K	79.7	2.62	1.33	0.0064	0.9224	2.79	0.0069	0.9983	0.0586	1.0851	0.7291
	135.7	4.30	3.29	0.0060	0.9939	4.49	0.0050	0.9999	0.0817	2.0842	0.8077
	153.1	4.64	2.73	0.0051	0.9873	5.05	0.0020	0.9972	0.1206	1.2763	0.8110

## Data Availability

The data generated and presented in this study are available on request from the corresponding author.
